# Biochemical characterization on muscle tissue of a novel biallelic *ACO2* mutation in an infant with progressive encephalopathy

**DOI:** 10.1002/jmd2.12400

**Published:** 2023-12-15

**Authors:** Federica Silvia Ricci, Serena Stanga, Mariarosa Mezzanotte, Cristina Marinaccio, Rossella D'Alessandro, Alessandra Somà, Stefano Sottemano, Alessandra Conio, Giovanni Morana, Marco Spada, Marina Boido, Tiziana E. Mongini

**Affiliations:** ^1^ Division of Child and Adolescent Neuropsychiatry, Department of Public Health and Pediatric Sciences University of Turin Turin Italy; ^2^ Department of Neuroscience “Rita Levi Montalcini” Neuroscience Institute Cavalieri Ottolenghi, University of Turin Turin Italy; ^3^ Division of Child and Adolescent Neuropsychiatry, Department of Child Care and Pathology Children Hospital “Regina Margherita” Turin Italy; ^4^ Pediatric Intensive Care Unit, Anesthesia, Resuscitation and Emergency Department University of Turin Turin Italy; ^5^ Neuroradiology Unit, Department of Neuroscience “Rita Levi Montalcini” University of Turin Turin Italy; ^6^ Division of Neurology 1, Department of Neuroscience “Rita Levi Montalcini” University of Turin Turin Italy

**Keywords:** *ACO2* gene, mitochondrial aconitase, neonatal neurometabolic disorder

## Abstract

The *ACO2* gene encodes the mitochondrial protein aconitate hydratase, which is responsible for catalyzing the interconversion of citrate into isocitrate in the tricarboxylic acid (TCA) cycle. Mitochondrial aconitase is expressed ubiquitously, and deficiencies in TCA‐cycle enzymes have been reported to cause various neurodegenerative diseases due to disruption of cellular energy metabolism and development of oxidative stress. We investigated a severe early infantile‐onset neurometabolic syndrome due to a homozygous novel variant in exon 13 of the *ACO2* gene. The in vitro pathogenicity of this variant of unknown significance was demonstrated by the loss of both protein expression and its enzymatic activity on muscle tissue sample taken from the patient. The patient presented with progressive encephalopathy soon after birth, characterized by hypotonia, progressive severe muscle atrophy, and respiratory failure. Serial brain magnetic resonance imaging showed progressive abnormalities compatible with a metabolic disorder, possibly mitochondrial. Muscle biopsy disclosed moderate myopathic alterations and features consistent with a mitochondriopathy albeit nonspecific. The course was characterized by progressive worsening of the clinical and neurological picture, and the patient died at 5 months of age. This study provides the first report on the validation in muscle from human subjects regarding in vitro analysis for mitochondrial aconitase activity. To our knowledge, no prior reports have demonstrated a correlation of phenotypic and diagnostic characteristics with in vitro muscle enzymatic activity of mitochondrial aconitase in humans. In conclusion, this case further expands the genetic spectrum of *ACO2* variants and defines a complex case of severe neonatal neurometabolic disorder.

**Synopsis**

Combining clinical, neuroradiological, genetic, and in vitro analysis to define a complex case of severe neonatal neurometabolic disorder and validate a homozygous novel variant in *ACO2* gene.

## 1 INTRODUCTION

The 18‐exons *ACO2* gene (OMIM *100850) encodes the mitochondrial protein aconitate hydratase (mHA, hereafter referred to as aconitase). It is a tricarboxylic acid (TCA) cycle iron–sulfur cluster protein that catalyzes the reversible isomerization of citrate to isocitrate via cis‐aconitate in the second step of the TCA cycle.^1^, ^2^ The TCA cycle's defects are linked to various neurodegenerative diseases, due to the disruption of cellular energy metabolism and development of oxidative stress.

Since the first two families were described in 2012, with involvement mainly of the cerebellum and retina,^2^ 19 families with *ACO2* mutations have been reported, expanding the spectrum of phenotypes from isolated optic atrophy^3^, ^4^ to hereditary spastic paraplegia^3^, ^5^ up to a severe infantile‐onset neurodegenerative disorder with hypotonia, severe psychomotor delay, epilepsy, progressive cerebellar and cortical atrophy, optic atrophy, and retinal degeneration (infantile cerebellar retinal degeneration, OMIM #614559).^1^ A recent screening of a European cohort of individuals with genetically unsolved inherited optic neuropathies, positioned *ACO2* as the third most frequently mutated gene in autosomal inherited optic neuropathies.^6^

Here, we report the case of a severe early infantile onset neurometabolic syndrome due to novel homozygous variant in exon 13 of the *ACO2* gene. The in vitro pathogenicity of this variant has been demonstrated by the loss of both protein expression and its enzymatic activity on muscle tissue sample taken from the patient.

This report was prepared in compliance with the regulations of the local ethics committee, and signed parental consent was obtained.

## 2 CASE REPORT

The patient was the first child of consanguineous parents (first cousins). She was born at term, without antenatal or perinatal problems, and a birth weight of 2500 g. At 15 days of age, she experienced her first episode of acute respiratory failure. At neurological examination, the infant had global severe hypotonia, decreased deep tendon reflexes, very poor antigravity movements, and absence of distal movements. No significant dysmorphisms were reported. Since the age of 3 months, the patient presented numerous episodes of apnea in sleep with a lowering of blood saturation to 84% and spontaneous recovery. She was unable to suck properly, with persistent failure to thrive. Ocular motility was characterized by the absence of fixation and tracking. The course was then characterized by a gradual evolution of severe muscle wasting, worsening of the hypotonia, and complete areflexia. The infant had bradycardia and apneas, requiring intubation and assisted ventilation. Due to recurring seizures, antiepileptic drugs were required. The severity of the neurological picture, with an evident state of distress and suffering, and the rapid and progressive worsening of the electroencephalogram (EEG) picture required the start of a continuous infusion therapy with midazolam, in combination therapy with morphine. The patient eventually fell into a deep coma and died at 5 months of life.

Regarding the diagnostic workup, EEGs revealed multifocal paroxysmal abnormalities and progressive deconstruction of electrical activity, consistent with nonspecific encephalopathy. Visual evoked potentials showed bilateral slowing of conduction. Somatosensory evoked potentials (median nerve stimulation) showed severe bilateral alteration of conduction, starting from the peripheral components, and bilateral absence of the N20‐P25 cortical complex. Electroneurography showed marked signs of mixed demyelinating and axonal motor and sensory polyneuropathy both in the upper and lower extremities. Echocardiography and abdominal ultrasound were normal. Extensive laboratory investigations, including glucose, lactate, ammonia, thyroid and adrenal function, creatine kinase, acylcarnitines, amino acids, and very long‐chain fatty acids were all normal.

**FIGURE 1:**
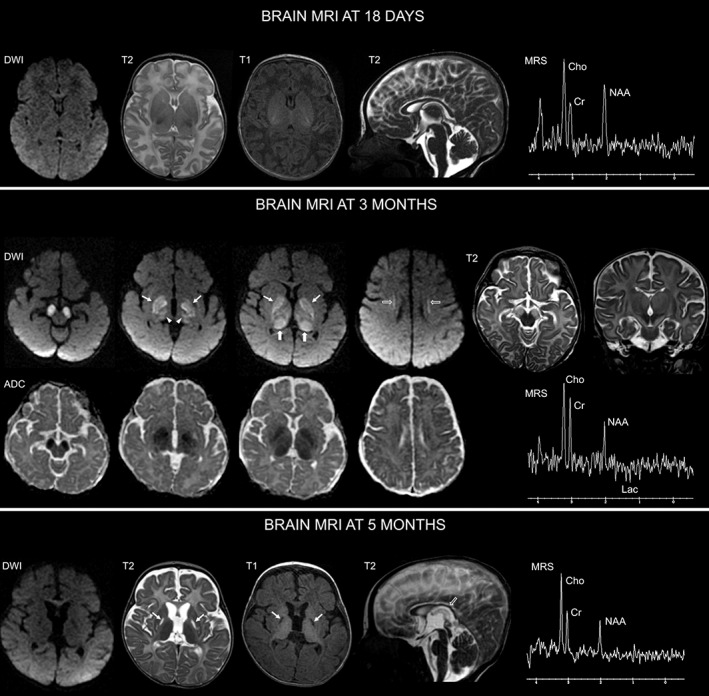
Brain MRI findings. At admission (18 days of life), axial diffusion‐weighted imaging (DWI) and T1‐weighted imaging, axial and sagittal T2‐weighted images, along with left thalamic single voxel magnetic resonance spectroscopy (MRS) with echo time (TE) of 144 ms, do not reveal definite structural and metabolic brain abnormality. Three months later, axial DWI and corresponding apparent diffusion coefficient (ADC) maps show restricted diffusivity involving the cerebral peduncles, subthalamic nuclei (arrowheads), globi pallidi (thin arrows) and thalami (thick arrows). Additional linear lesions with restricted diffusivity are demonstrated in the deep white matter, abutting the caudate nuclei (open arrows). Axial and coronal T2‐weighted images show increased signal in regions of restricted diffusivity. Single voxel MRS (TE 144 ms) of the left thalamic region shows a reduction of N‐acetylaspartate (NAA) and a lactate doublet (see also Figure S1). At 5 months, DWI, T1‐ and T2‐weighted images show reduced volume of the cerebral hemispheres, thalami, and basal ganglia (with cavitary lesion in both globi pallidi), no areas of restricted diffusivity, thinning of the posterior portion of the corpus callosum (open arrow) and ex‐vacuo dilatation of the ventricular system and subarachnoid spaces, in keeping with atrophic changes. Left thalamic single voxel MRS (TE 144 ms) shows further reduction of NAA without evidence of lactate.

The brain magnetic resonance imaging (MRI) performed at 18 months of age did not reveal any definite abnormalities (Figure 1). At 3 months of age, the brain MRI revealed bilateral and symmetric signal abnormalities hyperintense on T2‐weighted images and with restricted diffusivity on diffusion‐weighted imaging and corresponding apparent diffusion coefficient maps, involving the cerebral peduncles, subthalamic nuclei, thalami, globi pallidi, and with less extension the deep white matter. Localized magnetic resonance spectroscopy (MRS) of the left thalamic region demonstrated a reduction of N‐acetylaspartate (NAA) and a lactate doublet (Figures 1 and S1). An additional targeted MRS of the left centrum semiovale demonstrated a mild lactate peak. These overall findings were suggestive of a metabolic disorder, possibly mitochondrial (Leigh‐like MRI pattern). The last brain MRI performed at 5 months of age demonstrated diffuse atrophic changes in the brain, with a global delay in cerebral myelination compared with that expected for age. Localized MRS of the left thalamic region showed further reduction of NAA, without evidence of lactate (Figure 1).

The muscle biopsy revealed muscle tissue with moderate myopathic changes including fair variability in fiber caliber, inhomogeneity between fascicles, normally distributed nuclei, diffusely enlarged connective tissue, and normal vessels. It also revealed characteristics compatible with a mitochondriopathy albeit nonspecific (poor distinction of fiber types with oxidative enzymes: diffuse hyperactivity with NADH (reduced form of nicotinamide adenine dinucleotide) only a hint of distinction in types with LDH (lactate dehydrogenase, and inhomogeneity between fascicles with SDH (succinate dehydrogenase) and COX (cyclooxygenase) some with reduced activity). Immunohistochemistry showed a normal distribution of structural proteins.

Genetic tests included karyotype test (negative), comparative genomic hybridization (CGH) array (duplication of the 14q32.2 region with uncertain significance), and whole exome sequencing (WES), which revealed a homozygous variant in exon 13 of the ACO2 gene, c.1508G > C (p.Gly503Ala). The variant was also confirmed in the parents and is not reported in the dbSNP, gnomAD, or ClinVar databases. In silico analysis indicates that this variant could be deleterious (MutationTaster: disease‐causing; FATHMM‐MKL: damaging; Provean: damaging; DANN: 0.998, range 0–1, with 1 for maximum pathogenicity). Another missense variant at the same nucleotide position c.1508G > A (p.Gly503Glu) was previously reported as pathogenic in the Global Variome shared LOVD v.3.0 database.

## 3 IN VITRO VALIDATION

### 3.1 Mitochondrial proteins' enrichment from muscles' biopsies

To validate the WES results, enriched mitochondrial proteins have been extracted from muscle biopsy of the patient and an age‐matched control subject (from now on indicated as patient and control, respectively) according to the protocol of the assay Cayman Chemical, Item No. 705502. Mitochondrial proteins have been used for the enzymatic assay and Western blotting experiments.

### 3.2 Aconitase enzymatic activity test from muscles' biopsies

Enzymatic activity of mitochondrial aconitase has been measured by the aconitase assay (Cayman Chemical, Item No. 705502), as previously described^7^ on enriched mitochondrial protein extracted from the muscle biopsy of the patient and an age‐matched control subject. The rate at which nicotinamide adenine dinucleotide phosphate hydrogen (NADPH) is generated is proportional to the activity of aconitase.

From the enriched mitochondrial proteins extracted by the muscle biopsy of the patient and the control, we measured a marked reduction of about 66% of the aconitase activity in the muscles from the patient versus the control subject.

### 3.3 Western blotting for aconitase and mitochondrial markers

Western blotting experiments have been performed on enriched mitochondrial proteins extracted from the patient and an age‐matched control subject, according to standard protocols.^8^ After bicinchoninic acid (BCA) quantification, 7 μg of total protein lysates extracted were separated via sodium dodecyl sulfate‐polyacrylamide gel electrophoresis (SDS‐PAGE) with 4%–20% gradient gels (Biorad, Hercules, California, USA) and immunoblotted onto PVDF (polyvinilydene difluoride) membranes (GE Healthcare Life Sciences, Germany). Protein amounts were evaluated by using specific antibodies and normalized to Vinculin protein. The following primary antibodies were used to detect the proteins: antimitochondrial aconitase (1:1000; Cell Signaling, Massachusetts, USA), antimitochondrial import receptor subunit, Tom20 (1:1000; Cell Signaling, Massachusetts, USA), antimitochondrial oxidative phosphorylation system (OXPHOS), OXPHOS Cocktail (1:1500; Abcam, Cambridge, UK) and anti‐Vinculin (1:1000; Invitrogen, Massachusetts, USA). The membranes were incubated with secondary antibodies: for aconitase and Tom20; goat anti‐rabbit IgG‐HRP (1:6000) (Vector Laboratories, Burlingame, CA), for OXPHOS and Vinculin; goat anti‐mouse IgG‐HRP (1:6000) (Santa Cruz Biotechnology, USA). After secondary antibody incubation, the immunoreactivity was evaluated by using a chemiluminescence kit (Western Lightning Plus ECL). The fluorescence emitted was detected by a ChemiDoc XRS+ with Image Lab Software (Bio‐Rad). Data from WB quantification (Image Lab Software, BIO‐RAD, Italy) were normalized on levels of Vinculin bands.

**FIGURE 2:**
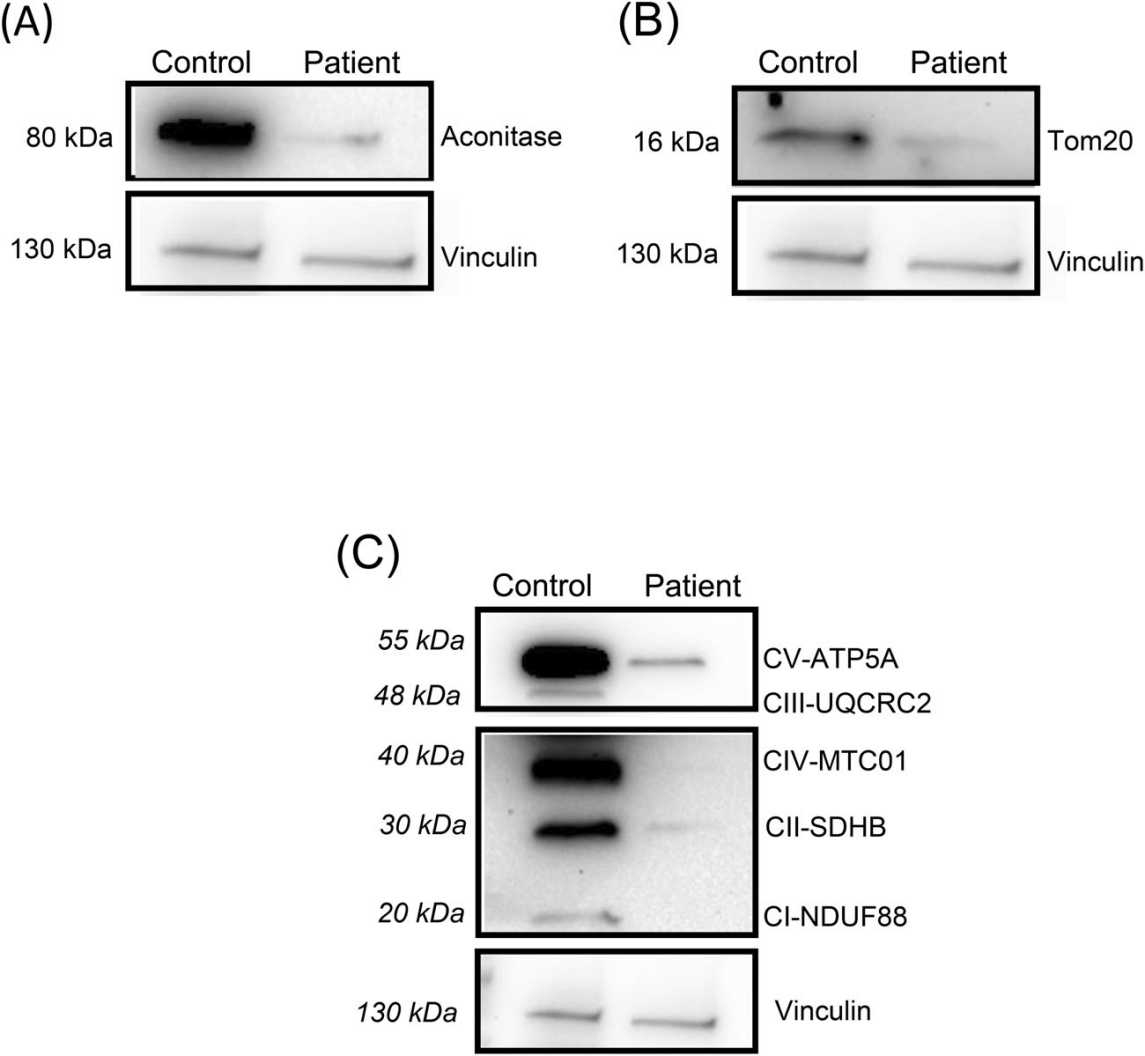
Quantification of mitochondrial proteins in muscles' biopsies. Western blotting analysis and quantification expressed as a percentage (%) of (A) mitochondrial aconitase, (B) mitochondrial import receptor subunit (Tom20), and (C) mitochondrial Oxidative Phosphorylation System (OXPHOS) in muscles' biopsies (control and patient) are shown. Data were normalized on Vinculin amount in the same samples (Image Lab 4.0.1 Sofware, Bio‐Rad, California, USA).

To investigate if the strong reduction in the enzymatic activity of mitochondrial aconitase was due to a reduction of its protein levels or to mitochondrial content, we performed the western blotting analysis on enriched mitochondrial proteins extracted from the muscle biopsy of the patient and an age‐matched control subject. The western blotting showed a significant reduction of mitochondrial aconitase protein levels of about 97% in the patient compared with the control subject (Figure 2A). For the mitochondrial content, we analyzed the expression of Tom20 (translocase of the outer membrane 20), a multisubunit protein complex of the outer mitochondrial membrane (OMM), involved in the import of nucleus‐encoded precursor proteins across the OMM. Western blotting revealed a strong reduction of about 91% of the protein amount in the patient compared with the control subject (Figure 2B). Since we found alterations in aconitase enzymatic activity, and since it is known that the TCA cycle is in direct relation with respiratory chain (OXPHOS) for the maintenance of balanced cellular metabolism, we evaluated the expression of the five complexes of the respiratory chain using the OXPHOS cocktail of antibodies on enriched mitochondrial proteins extracted from muscle biopsies. The results showed a statistically significant decrease in the expression of all the five complexes of the respiratory chain in the patient compared with the control (Figure 2C).

## 4 DISCUSSION

A homozygous novel variant in exon 13 of the ACO2 gene, which encodes mitochondrial aconitase, was identified in a case of severe early infantile onset neurometabolic syndrome.

As well as the cytoplasmic form, mitochondrial aconitase is ubiquitously expressed and catalyzes the interconversion of citrate into isocitrate in the TCA cycle.^9^ The TCA cycle has a crucial role in the mitochondrial metabolism, generating reduced power in the form of NADH and FADH2.^10^ In case of stressful cellular conditions, aconitase undergoes posttranslational modifications, resulting in loss of activity.^11^ In addition to the main role in the TCA cycle, aconitase is involved in mitochondrial DNA stabilization with pathophysiological mechanisms including cellular energy metabolism disruption and mitochondrial DNA depletion when deficient in humans.^12^, ^13^ Aconitase deficiency may also cause neurotoxicity by the accumulation of glutamate and free radical species.^13^

Deficiencies in the TCA‐cycle enzymes (e.g., fumarate hydratase, succinate dehydrogenase, and succinyl‐CoA synthetase) have been reported to cause multisystem disorders, including early‐onset encephalopathies.^14^ A neurometabolic early onset syndrome with *ACO2* mutations has been fully described, with typical onset between 2 and 6 months of age, and clinical features including trunk hypotonia, athetosis, epilepsy, and ophthalmologic abnormalities. In affected individuals, MRI shows progressive cerebral and cerebellar degeneration.^1^ Although a clear genotype–phenotype correlation has not been demonstrated, according to some reports the amount of residual aconitase activity could mitigate the clinical severity.^5^

Diagnosing aconitase deficiency in clinical practice can be challenging due to the overlap of clinical features with other neurometabolic or neurodegenerative disorders, the lack of specific mitochondrial biochemical abnormalities such as lactic acidosis, and the delayed evidence of MRI abnormalities (diffuse cerebellar atrophy and cortical atrophy) compared with severe clinical features.^1^ Our patient presented with a progressive encephalopathy (hypotonia, hyporeflexia, very poor antigravity movements, disordered ocular motility, persistent failure to thrive, and respiratory failure) soon after birth but without specific biochemical markers. The first brain MRI, performed 18 days after birth, was normal. However, the second MRI, performed at 3 months of age showed a Leigh‐like pattern. This MRI/MRS finding in ACO2 mutations was previously unreported and oriented us toward a mitochondrial disorder. A muscle biopsy disclosed moderate myopathic alterations and features consistent with a mitochondriopathy albeit nonspecific. The biochemical validation in muscle tissue of the homozygous variant in exon 13 of the *ACO2* gene c.1508G > C (p.Gly503Ala), detected by WES, allowed for the diagnosis and genetic counseling of the parents. As reported by Sharkia et al.^15^ in a series of 16 patients, also in our case enzymatic activities of the mitochondria were not intact and helped orientate the diagnosis. The last brain MRI, performed a few days before patient's death, documented the progressive evolution of the known picture with cavitating areas and atrophic cerebral changes.

Regarding in vitro analysis, to our knowledge, this is the first report on the validation of mitochondrial aconitase activity in muscle from human subjects. Although validation in fibroblasts derived from patients has been reported,^3^, ^13^ no other case report to date has demonstrated the correlation of phenotypic and diagnostic features with the muscular in vitro enzymatic activity of aconitase in humans.

In conclusion, our case report further expands the genetic spectrum of *ACO2* variants. The combination of clinical features, serial neuroradiological follow‐up, next‐generation sequencing, and demonstration of loss of protein expression and its enzymatic activity in muscle tissue has contributed to the accurate definition of this complex case of severe neonatal neurometabolic disorder.

## AUTHOR CONTRIBUTIONS

Federica Silvia Ricci, Serena Stanga, Alessandra Somà, Stefano Sottemano, Giovanni Morana, Marina Boido, and Tiziana E. Mongini were involved in planning, conducting, and reporting the study. Mariarosa Mezzanotte, Cristina Marinaccio, Rossella D'Alessandro, Alessandra Conio, and Marco Spada were involved in conducting the study.

## CONFLICT OF INTEREST STATEMENT

The authors declare no conflicts of interest.

## ETHICS STATEMENT

All procedures followed were in accordance with the ethical standards of the responsible committee on human experimentation (institutional and national) and with the Helsinki Declaration of 1975, as revised in 2000.

## INFORMED CONSENT

Informed consent was obtained from all patients for being included in the study.

## Supporting information


**Figure S1.** Single voxel MRS (TE 144 ms) data performed at 3 months processed by LCModel program. Localized MRS of the left thalamic region demonstrates the presence of lactate and NAA reduction. Localized MRS of the left centrum semiovale shows a mild lactate peak.

## Data Availability

The data that support the findings of this study are available from the corresponding author upon reasonable request.

## References

[1] Park JS , Kim MJ , Kim SY , et al. Novel compound heterozygous ACO2 mutations in an infant with progressive encephalopathy: a newly identified neurometabolic syndrome. Brain Dev. 2020;42(9):680‐685. 32713659 10.1016/j.braindev.2020.07.003

[2] Spiegel R , Pines O , Ta‐Shma A , et al. Infantile cerebellar‐retinal degeneration associated with a mutation in mitochondrial aconitase, ACO2. Am J Hum Genet. 2012;90(3):518‐523. 22405087 10.1016/j.ajhg.2012.01.009 PMC3309186

[3] Metodiev MD , Gerber S , Hubert L , et al. Mutations in the tricarboxylic acid cycle enzyme, aconitase 2, cause either isolated or syndromic optic neuropathy with encephalopathy and cerebellar atrophy. J Med Genet. 2014;51(12):834‐838. 25351951 10.1136/jmedgenet-2014-102532

[4] Kelman JC , Kamien BA , Murray NC , Goel H , Fraser CL , e Grigg JR . A sibling study of isolated optic neuropathy associated with novel variants in the ACO2 gene. Ophthalmic Genet. 2018;39(5):648‐651. 30118607 10.1080/13816810.2018.1509353

[5] Bouwkamp CG , Afawi Z , Fattal‐Valevski A , et al. *ACO2* homozygous missense mutation associated with complicated hereditary spastic paraplegia. Neurol Genet. 2018;4(2):e223. 29577077 10.1212/NXG.0000000000000223 PMC5863690

[6] Charif M , Gueguen N , Ferré M , et al. Dominant *ACO2* mutations are a frequent cause of isolated optic atrophy. Brain Commun. 2021;3(2):fcab063. 34056600 10.1093/braincomms/fcab063 PMC8152918

[7] Wyart E , Hsu MY , Sartori R , et al. Iron supplementation is sufficient to rescue skeletal muscle mass and function in cancer cachexia. EMBO Rep. 2022;23(4):e53746. 35199910 10.15252/embr.202153746 PMC8982578

[8] Opsomer R , Contino S , Perrin F , et al. Amyloid precursor protein (APP) controls the expression of the transcriptional activator neuronal PAS domain protein 4 (NPAS4) and synaptic GABA release. eNeuro. 2020;7(3):ENEURO.0322‐19.2020. 10.1523/ENEURO.0322-19.2020 PMC7262005 32327470

[9] Slaughter CA , Hopkinson DA , e Harris H . The distribution and properties of aconitase isozymes in man. Ann Hum Genet. 1977;40(4):385‐401. 879710

[10] Raimundo N , Baysal BE , e Shadel GS . Revisiting the TCA cycle: signaling to tumor formation. Trends Mol Med. 2011;17(11):641‐649. 21764377 10.1016/j.molmed.2011.06.001 PMC3205302

[11] Lushchak OV , Piroddi M , Galli F , e Lushchak VI . Aconitase post‐translational modification as a key in linkage between Krebs cycle, iron homeostasis, redox signaling, and metabolism of reactive oxygen species. Redox Rep. 2014;19(1):8‐15. 24266943 10.1179/1351000213Y.0000000073 PMC6837700

[12] Chen XJ , Wang X , Kaufman BA , Butow RA . Aconitase couples metabolic regulation to mitochondrial DNA maintenance. Science. 2005;307(5710):714‐717. 15692048 10.1126/science.1106391

[13] Sadat R , Barca E , Masand R , et al. Functional cellular analyses reveal energy metabolism defect and mitochondrial DNA depletion in a case of mitochondrial aconitase deficiency. Mol Genet Metab. 2016;118(1):28‐34. 26992325 10.1016/j.ymgme.2016.03.004 PMC4833660

[14] Munnich A . Casting an eye on the Krebs cycle. Nat Genet. 2008;40(10):1148‐1149. 18818715 10.1038/ng1008-1148

[15] Sharkia R , Wierenga KJ , Kessel A , et al. Clinical, radiological, and genetic characteristics of 16 patients with ACO2 gene defects: delineation of an emerging neurometabolic syndrome. J Inherit Metab Dis. 2019;42(2):264‐275. 30689204 10.1002/jimd.12022

