# Supplementary figures and images for: Biochemical characterization on muscle tissue of a novel biallelic ACO2 mutation in an infant with progressive encephalopathy
Source: JIMD Rep. 2023 Dec 15;65(1):3–9. doi: 10.1002/jmd2.12400 (PMC10764196; doi:10.1002/jmd2.12400)

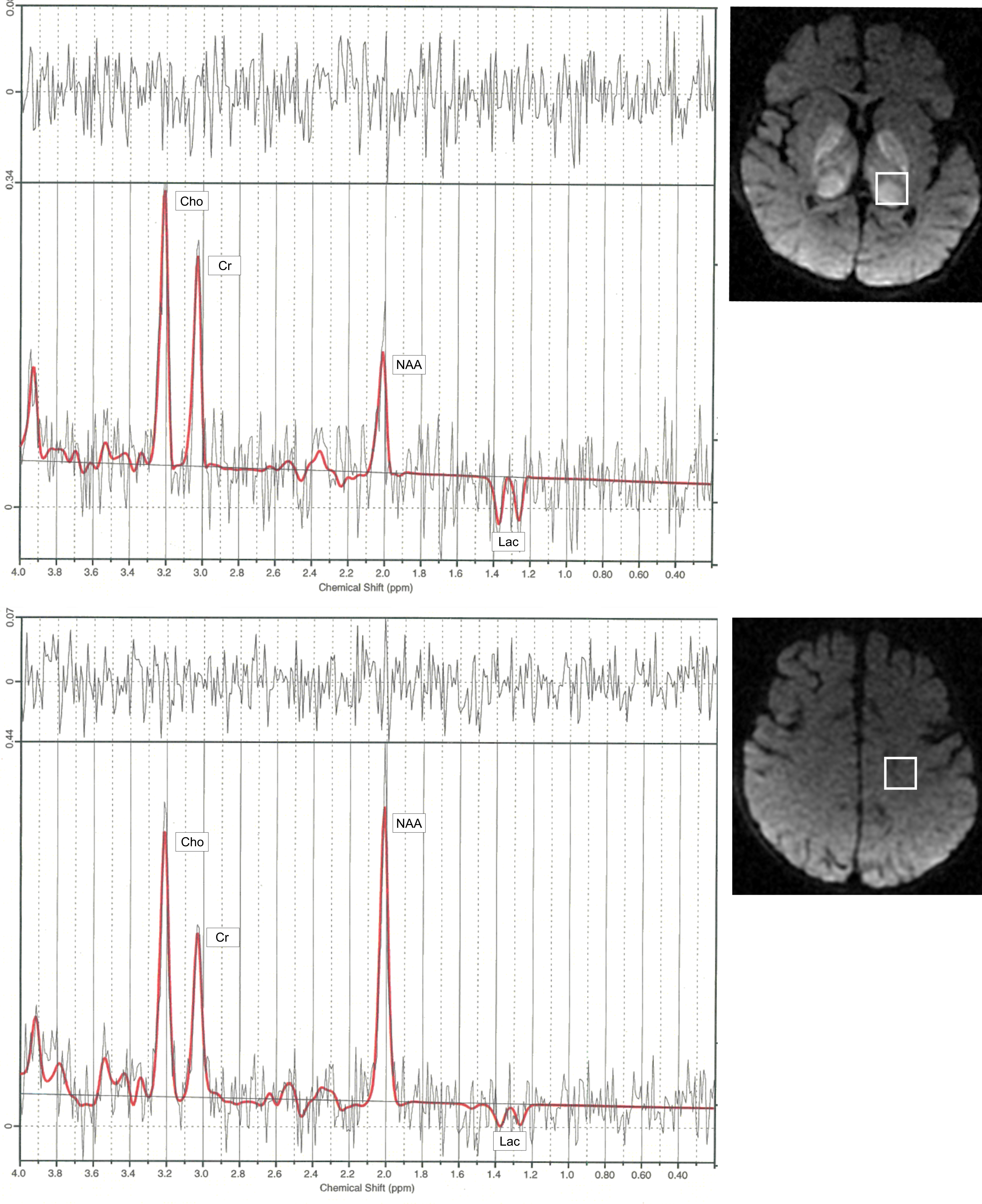

Supplement: Supplementary file 1 — Figure S1. Single voxel MRS (TE 144 ms) data performed at 3 months processed by LCModel program. Localized MRS of the left thalamic region demonstrates the presence of lactate and NAA reduction. Localized MRS of the left centrum semiovale shows a mild lactate peak. [file JMD2-65-3-s001.tiff]
